# Cardiovascular organ damage in type 2 diabetes mellitus: the role of lipids and inflammation

**DOI:** 10.1186/s12933-019-0865-6

**Published:** 2019-05-10

**Authors:** Michaela Kozakova, Carmela Morizzo, Isabel Goncalves, Andrea Natali, Jan Nilsson, Carlo Palombo

**Affiliations:** 1 Department of Clinical and Experimental Medicine, University of Pisa, Via Savi 10, 56126 Pisa, Italy; 2 Department of Surgical, Medical Molecular Pathology and Critical Care Medicine, University of Pisa, Via Savi 10, 56126 Pisa, Italy; 3 Department of Clinical Sciences Malmö, Lund University, Jan Waldenströms gata 35, 20502 Malmö, Sweden

**Keywords:** Diabetes mellitus, Interleukins, Matrix-metalloproteinase, High density lipoprotein cholesterol, Arterial stiffness, Left ventricular mass

## Abstract

**Background:**

The relationship between dyslipidemia, inflammation and CV organ damage in type 2 diabetes mellitus (T2DM) is complex. Insulin resistance and inflammatory cytokines interleukins (ILs) increase plasma triglycerides (TG). ILs also up-regulate expression of matrix-metalloproteinases (MMPs) that, together with TG, decrease high density lipoprotein cholesterol (HDL) levels. High TG, low HDL, increased ILs and MMPs trigger structural and functional changes in different parts of cardiovascular (CV) system. To understand better the role of lipids and inflammation in CV organ damage, the present study investigated the inter-relationships between lipids, ILs and MMPs, as well as the associations of lipids, ILs and MMPs with various CV measures, both in diabetic and non-diabetic population (nonT2DM).

**Methods:**

In T2DM patients (N = 191) and nonT2DM subjects (N = 94) were assessed carotid intima-media thickness (cIMT) and inter-adventitial diameter (IADiam), carotid wave speed (ccaWS), carotid-femoral pulse wave velocity (cfPWV), left ventricular (LV) mass, LV systolic (s′) and early diastolic (e′) longitudinal velocities of mitral annulus, together with glycemic control, lipid profile, IL-6, IL-18 and MMP-12.

**Results:**

T2DM patients, as compared to nonT2DM subjects, had significantly higher plasma levels of IL-6, IL-18, MMP-12 and lower HDL (*P *< 0.05–0.0001). They had also higher cIMT, IADiam, ccaWS, cfPWV and LV mass, and lower e′ velocity (*P *< 0.005–0.0001). Both in T2DM patients and nonT2DM subjects, MMP-12 increased with IL-6 (r = 0.43 and 0.39; *P *< 0.0001) and IL-18 (r = 0.32 and 0.42; *P *< 0.0001), and HDL decreased with MMP-12 (r = − 0.29 and − 0.42; *P *< 0.0001). In both populations, MMP-12 was directly associated with IADiam, ccaWS, cfPWV and LV mass (r = 0.42, 0.32, 0.26 and 0.29; *P *< 0.0001 in T2DM patients, and r = 0.39, 0.28, 0.32 and 0.27; *P *< 0.01–0.0001 in nonT2DM subjects). In multivariate analysis, MMP-12 remained independently related to IADiam, ccaWS, cfPWV and LV mass in T2DM patients, and to IADiam only in nonT2DM subjects.

**Conclusions:**

This cross-sectional study demonstrated a direct association between ILs and MMP-12, as well as an inverse association between MMP-12 and HDL, both in T2DM patients and in nonT2DM subjects. In T2DM patients, who had higher levels of ILs and MMP-12, the latter was independently related to several structural and functional markers of preclinical CV organ damage.

**Electronic supplementary material:**

The online version of this article (10.1186/s12933-019-0865-6) contains supplementary material, which is available to authorized users.

## Background

Type 2 diabetes mellitus (T2DM) is associated with increased cardiovascular (CV) morbidity and mortality [1] and a major pathophysiologic mechanism linking T2DM to CV risk is diabetic dyslipidaemia, characterized by high triglycerides (TG) and low high density lipoprotein cholesterol (HDL) plasma levels [2]. The two lipids are closely connected in T2DM, since the insulin resistance-induced increase in plasma TG triggers the catabolism of HDL, and consequently, a specific role of the two lipids in CV organ damage is not clearly understood [3]. Moreover, T2DM is a chronic low-grade inflammation state [4], and inflammatory cytokines may also influence plasma lipids levels [5, 6]. Interleukins (ILs) stimulate hepatic secretion of TG [6] and up-regulate expression and activity of matrix-metalloproteinases (MMPs) [7], the endopeptidases that decrease HDL levels through degradation of apolipoprotein A-I [8]. Yet, interleukins and MMPs may not only modify lipid profile but also directly damage CV system [9–13].

It is evident that the relationship between lipids, inflammation and CV organ damage in T2DM patients is complex. Number of studies have shown the associations of TG, HDL, ILs or MMPs with vascular and cardiac alterations [9, 12–17], yet the majority of these studies have evaluated the impact of a single metabolic abnormality on a single CV measure, without taking into consideration the relationships between lipids and inflammation and without considering that the heart and large arteries are closely anatomically and functionally linked and that the changes in vascular tree have impact on the structure and function of the heart.

To understand better the role of dyslipidemia and inflammation in CV organ damage and CV risk of T2DM patients, the present study evaluated the inter-relationships between TG, HDL, ILs and MMP-12, as well as the associations of lipids, ILs and MMP-12 with different measures of CV structure and function, both in T2DM patients and in subjects free of diabetes (nonT2DM).

## Methods

### Study population

Study population consists of 191 T2DM patients and 94 nonT2DM subjects of similar age and comparable prevalence of CV disease. All subjects were referred for a complete CV examination to the Clinic for Cardiometabolic Risk Prevention of the Department of Surgical and Medical Pathology, University of Pisa between December 2010 and April 2013. Diagnosis of T2DM was based on plasma glucose criteria (fasting glucose ≥ 7 mmol/L or 2-h glucose during oral-glucose tolerance test ≥ 11.1 mmol/L) or HbA1c ≥ 6.5% [18]. Two abnormal test results from the same sample or in two separate test samples were required to confirm the diagnosis.

Twenty-one T2DM patients were treated by diet, 135 by oral antidiabetic drugs, 22 by a combination of oral antidiabetics and insulin and 13 by insulin only. Antagonists of the renin–angiotensin–aldosterone system (ACE inhibitors and ARBs) were the most frequently used anti-hypertensive agent, followed by beta-blockers, both in T2DM patients and nonT2DM subjects.

### Study protocol

The protocol of the study followed the principles of the Declaration of Helsinki and was approved by the institutional ethics committee “Comitato Etico di Area Vasta Nord Ovest” (Reference Number: 3146/2010). All subjects gave their informed consent to participate.

#### Vascular examination

All study subjects underwent carotid ultrasound and measurement of carotid-femoral pulse wave velocity (cfPWV). Vascular examination was performed in the afternoon, 3 h after a light meal, in a quiet room with a stable temperature of 22°, after resting comfortably for at least 15 min in the supine position. All subjects were asked to abstain from cigarette smoking, caffeine and alcohol consumption and vigorous physical activity for 24 h.

CfPWV was measured according to current guidelines using the Complior device (Alam Medical, Vincennes, France). In our laboratory, intra- and inter-individual variability of cfPWV measurement are 4.3 ± 2.8% and 5.1 ± 2.9%, respectively. Increase in cfPWV was defined as cfPWV higher than 90th age- and BP-specific percentiles of cfPWV observed in a reference population of 11.092 subjects [19].

Carotid ultrasound was performed by a single operator (CM) on the right common carotid artery using an ultrasound scanner equipped with a 10 MHz linear probe (MyLab 70, Esaote, Genova, Italy) and implemented with a previously validated radiofrequency-based tracking of arterial wall (QIMT^®^ and QAS^®^) that allows an automatic and real-time determination of far-wall cIMT, IADiam and carotid distension with a high spatial and temporal resolution (sampling rate of 550 Hz on 32 lines). From the distension curves a local one-point carotid wave speed (ccaWS) was calculated applying the Bramwell–Hill equation that relates the propagation velocity to arterial distensibility as previously described [20]. Increase in cIMT was defined as IMT higher than 90th age- and sex-specific percentiles of cIMT observed in a reference population of 1.993 men and women [21]. All radiofrequency-derived measures were averaged over 6 consecutive cardiac beats and the values used for statistical analysis represent a mean of three consecutive acquisitions.

#### Echocardiographic examination

Left ventricle (LV) mass was measured by echocardiography (MyLab 70, Esaote, Genova, Italy, equipped with a 1.8–3.5 MHz, phased-array probe). LV inner diameter and wall thickness were measured in end-diastole and end-systole in M-mode images, and LV mass was calculated by Penn formula [22]. LV mass index was calculated as LV mass per height^2.7^ and LV hypertrophy was defined as LV mass index > 51 g/m^2.7^ in both men and women [23]. End-systolic wall stress was calculated as recommended [24]. Stroke volume was assessed as the product of aortic valve cross-sectional area and transaortic flow-velocity time integral.

Systolic and diastolic LV longitudinal velocities at mitral annular level, both at septal and lateral sides, were measured by color-guided pulsed-wave tissue Doppler in the apical four-chamber view. The sample volume was placed at the junction of the LV wall with the mitral annulus, and the cursor was aligned so that the angle of incidence between the Doppler beam and the longitudinal motion of the LV was as close as possible to 0°. From spectral traces, peak longitudinal velocity during systole (s′) and during early diastolic filling (e′) were measured and averaged over 5 consecutive cardiac cycles [25]. Reported values represent an average of septal and lateral sides. The intra-individual variability of tissue Doppler measurements in our laboratory is 4.7 ± 3.5% and 5.8 ± 4.3% for s′ and e′ velocity, respectively.

#### Medical history, physical and instrumental examination

A standardized medical history, physical examination and resting ECG and echocardiography were performed in all subjects. CV disease was defined as coronary artery disease and unstable angina, infarction, heart failure, stroke or peripheral macrovascular disease. Height and weight were obtained and body mass index (BMI) was calculated. Waist circumference was measured as the narrowest circumference between the lower rib margin and anterior superior iliac crest. Office brachial BP was measured twice during two different visits in a seated patient, using a standard mercury sphygmomanometer; regular or large adult cuffs were used, depending on patient arm circumference. The mean value of the two measurements was calculated and used for statistical analysis. Smoking habit was evaluated as never, past and current smoker.

#### Analytical procedures

Plasma levels of low density lipoprotein cholesterol (LDL), HDL, TG and glucose were determined within 1 week of CV examination by standard methods on a Roche-Modular Autoanalyzer (Milan, Italy). Glycosylated hemoglobin (HbA1c) was measured by high-performance liquid chromatography and standardized against Diabetes Control and Complications Trial (DCCT ) standard. Plasma levels of biomarkers reflecting inflammation (IL-6 and IL-18) and extracellular matrix proteolysis (MMP-12) were analyzed by the Proximity Extension Assay (PEA) technique using the Proseek Multiplex CVD^96×96^ reagents kit (Olink Bioscience, Uppsala, Sweden) at the Clinical Biomarkers Facility, Science for Life Laboratory, Uppsala as previously described [9, 26]. All samples were analyzed in the same run. Data analysis was performed by a preprocessing normalization procedure using Olink Wizard for GenEx (Multid Analyses, Sweden). Values are presented as arbitrary units (AU). Data regarding intra- and inter-assays variations as well as general calibrator curves to calculate the approximate concentrations are available on the OLINK homepage (http://www.olink.com).

#### Statistical analysis

Data are expressed as mean ± SD, categorical data as percentages. Variables with skewed distribution were summarized as median [interquartile range], and were logarithmically transformed for parametric statistical analysis. ANOVA was used to compare continuous variables, and a χ^2^ test for categorical variables. The univariate relationships between the outcome variables and continuous variables were assessed by Pearson (r) correlation coefficient. Multiple linear regression analyses (controlled for age, sex, body size, BP, heart rate, smoking habit, BP-lowering and lipid-lowering therapy and, in T2DM patient, also for diabetes duration and antidiabetic treatment) with backward stepwise removal were used to identify the independent associations of outcome variables with their significant univariate correlates. Statistical tests were two-sided, and significance was set at a value of *P *< 0.05. Statistical analysis was performed by JMP software, version 3.1 (SAS Institute Inc., Cary, NC, USA).

## Results

### Characteristics of the study population

Characteristics of T2DM patients and nonT2DM subjects are reported in Table 1. As compared to nonT2DM subjects, T2DM patients were on average 3 years older and had significantly higher body size, systolic BP and heart rate, plasma levels of fasting glucose, Hb1AC, ILs and MMP-12, and lower plasma HDL levels. The prevalence of BP-lowering and lipid-lowering therapy, but not that of CV events, was higher in T2DM. T2DM patients had higher cIMT, IADiam, ccaWS, cfPWV, LV mass and lower e′ velocity. The prevalence of established organ damage (increased cIMT, cfPWV and LV hypertrophy) was also higher in T2DM.

**Table 1:** Characteristics of study population (mean ± SD/median [IQR])

	T2DM patients	nonT2DM subjects	
Gender (M:F)	148:43	66:28	0.20
Age (years)	65 ± 7	62 ± 8	< 0.0001
T2DM duration (years)	6 [11]		
Body weight (kg)	84 ± 15	79 ± 12	< 0.005
BMI (kg/m^2^)	29.0 ± 4.5	27.2 ± 3.6	< 0.001
Waist circumference (cm)	106 ± 12	98 ± 11	< 0.0001
Systolic BP/diastolic BP (mmHg)	135 ± 19/77 ± 10	128 ± 15/78 ± 10	< 0.05^a^
Heart rate (bpm)	66 ± 11	60 ± 9	< 0.0001
LDL-cholesterol (mmol/L)	2.67 ± 0.85	2.86 ± 0.94	0.09
HDL-cholesterol (mmol/L)	1.27 ± 0.28	1.35 ± 0.35	< 0.05
Triglycerides (mmol/L)	1.32 [0.82]	1.25 [0.61]	0.08
Fasting glucose (mmol/L)	7.4 ± 1.8	4.9 ± 0.6	< 0.0001
HbA1c (%)	7.0 [1.4]	5.7 [0.4]	< 0.0001
hsCRP (mg/dL)	0.15 [0.26]	0.17 [0.19]	0.40
IL-6 (AU)	36 [25]	29 [25]	< 0.01
IL-18 (AU)	1314 [637]	1140 [557]	0.005
MMP-12 (AU)	139 [96]	98 [58]	< 0.0001
BP-lowering therapy (%)	73.3	52.1	< 0.0005
Lipid-lowering therapy (%)	62.8	50.0	< 0.05
Current smoking (%)	31.1	34.0	0.57
CV events (%)	49.2	40.4	0.44^a^
cIMT (μm)	782 ± 129	668 ± 139	< 0.01^a^
cIMT > 90th percentile (%)	33.5	18.1	0.005
IADiam (mm)	8.52 ± 0.91	8.05 ± 0.92	< 0.005^a^
ccaWS (m/s)	8.9 ± 1.8	7.9 ± 1.5	0.001^a^
cfPWV (m/s)	11.4 ± 2.4	9.3 ± 2.0	< 0.0001^a^
cfPWV > 90th percentile (%)	27.2	4.3	< 0.0001
LV mass (g)	225 ± 54	199 ± 52	< 0.0005
LV hypertrophy (%)	51.8	29.8	< 0.001
s′ velocity (cm/s)	8.6 ± 1.3	8.8 ± 1.6	0.28
e′ velocity (cm/s)	8.7 ± 1.4	9.7 ± 2.0	< 0.0005^a^
Stroke volume (mL)	81 ± 16	70 ± 19	< 0.0001
End-systolic wall stress (*10^3^ dynes/cm^2^)	72 ± 24	69 ± 29	0.44

^a^After adjustment for age

### Inter-relationships between lipids, ILs and MMP-12

**Fig. 1:**
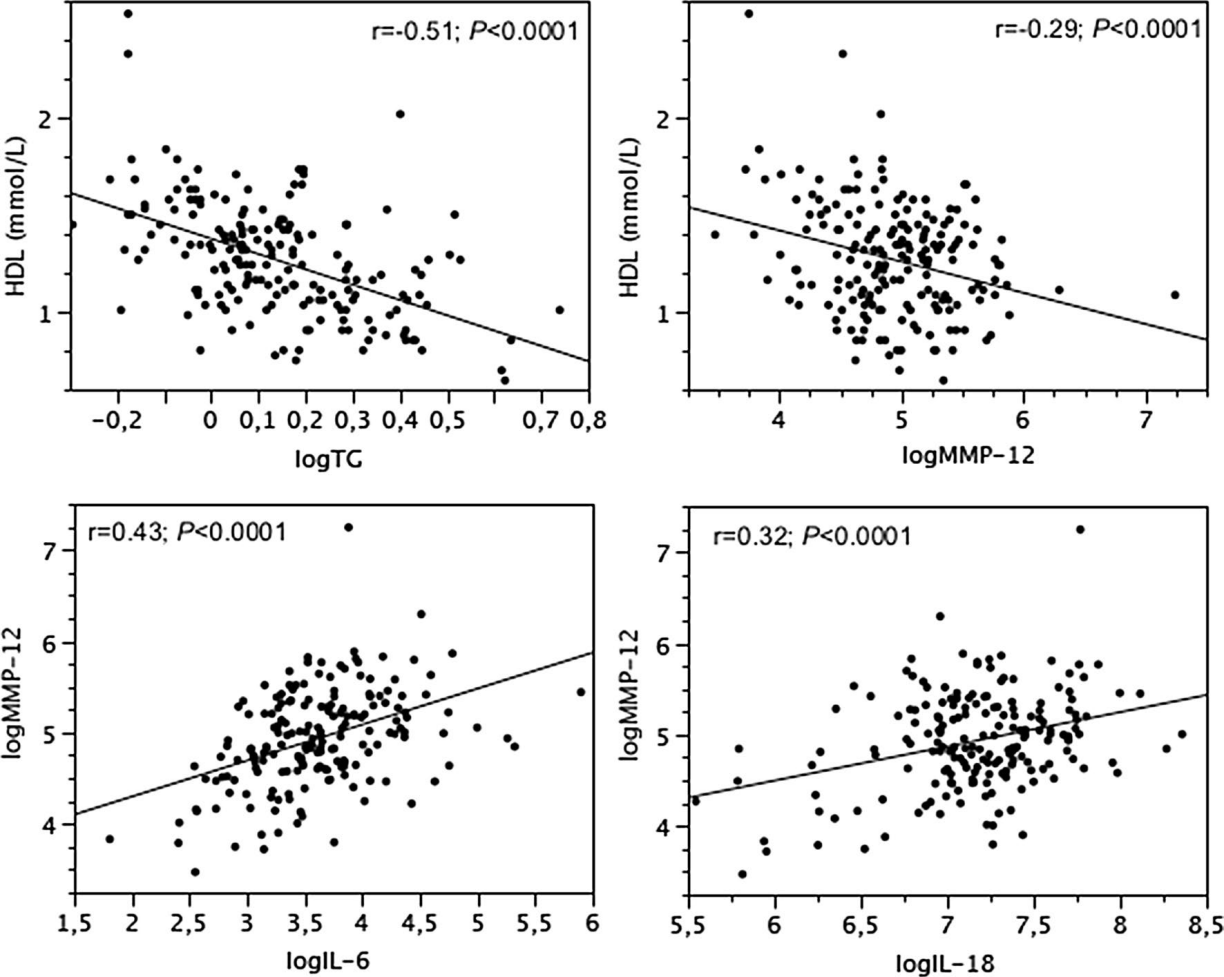
Relationships between HDL, TG and MMP-12, and relationships between MMP-12, IL-6 and IL-18 in T2DM patients

**Fig. 2:**
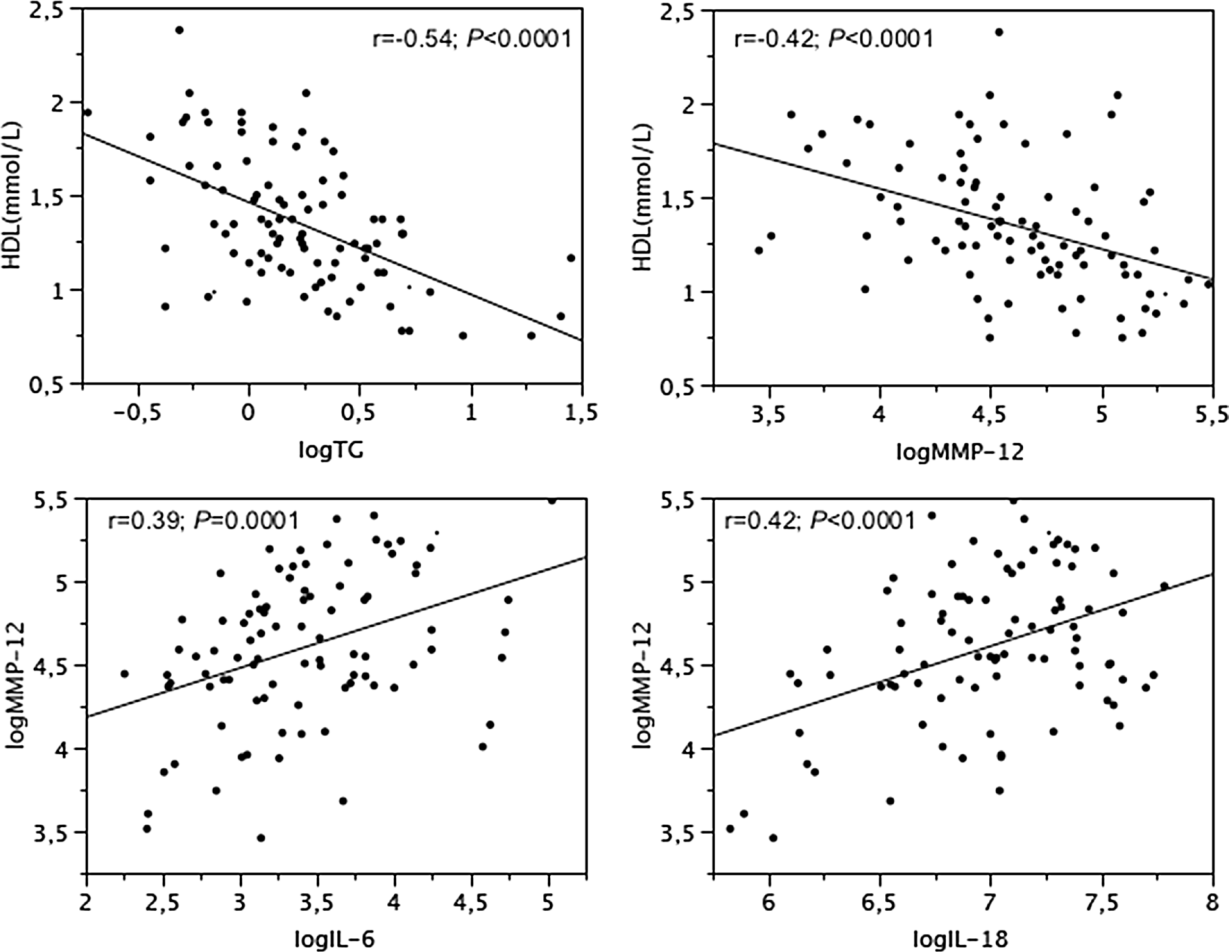
Relationships between HDL, TG and MMP-12, and relationships between MMP-12, IL-6 and IL-18 in nonT2DM subjects

In both T2DM patients (Fig. 1) and nonT2DM subjects (Fig. 2), HDL decreased with TG and MMP-12, and MMP-12 increased with IL-6 and IL-18. In T2DM only, TG increased with fasting plasma glucose levels (r = 0.35; *P *< 0.0001). Fasting glucose and HbA1c was not related to ILs in either population.

### CV measures, lipids, ILs and MMP-12

**Fig. 3:**
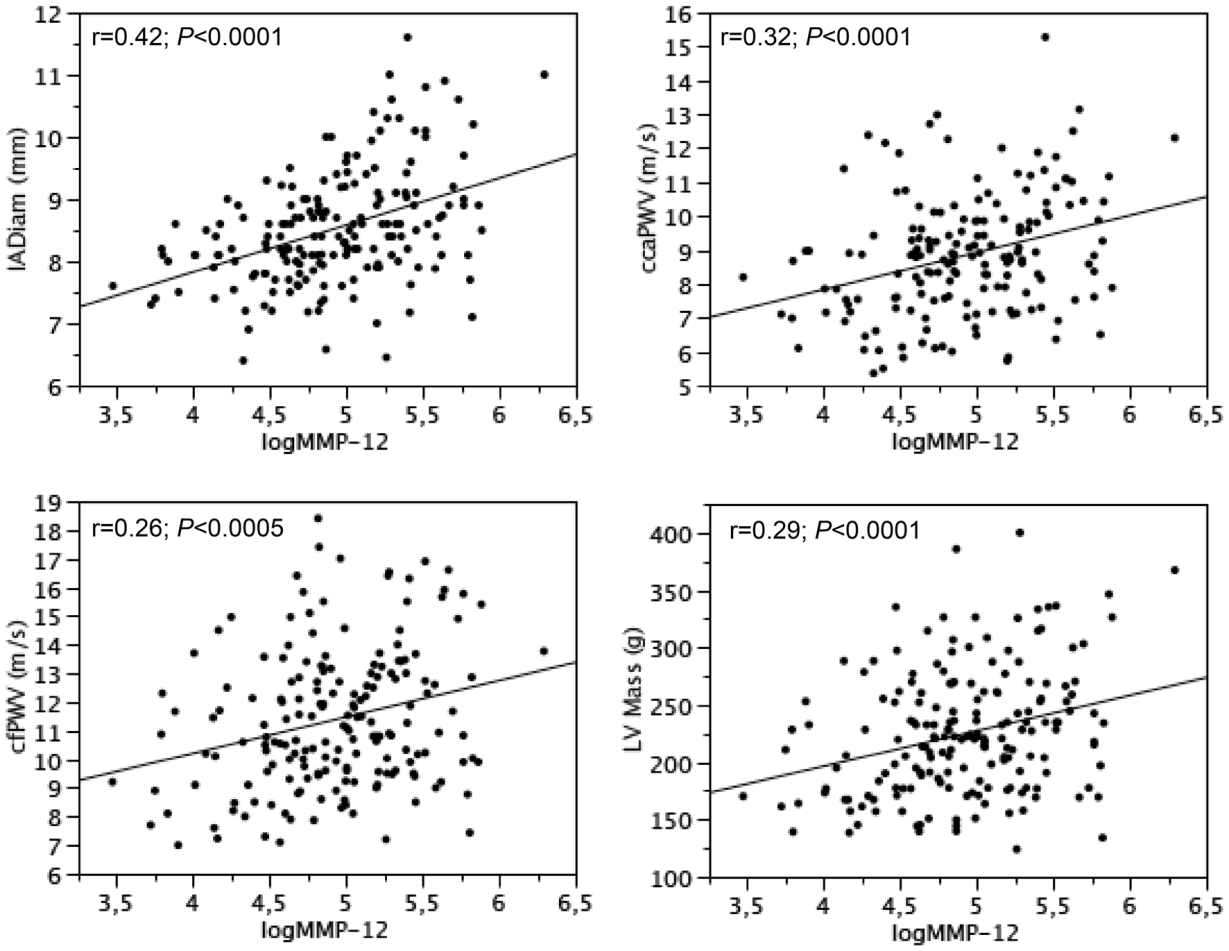
Relationships between MMP-12 and carotid diameter, carotid wave speed, carotid-femoral pulse wave velocity and LV mass in T2DM patients

In T2DM patients, cIMT, IADiam, ccaWS, cfPWV and LV mass were inversely related to HDL (r = − 0.31, − 0.26, − 0.24, − 0.21 and − 0.23; *P *< 0.005–0.0001, and IADiam, ccaWS, cfPWV and LV mass were directly related to MMP-12 (Fig. 3). LV mass was also directly related to fasting glucose and IL-6 (r = 0.20 and 0.25; *P *< 0.01 and < 0.001). E′ velocity of mitral annulus was inversely related to fasting glucose (r = − 0.18; *P *< 0.05) and directly to HDL (r = 0.24; *P *< 0.001), whereas s′ velocity was not related to any metabolic parameter.

**Fig. 4:**
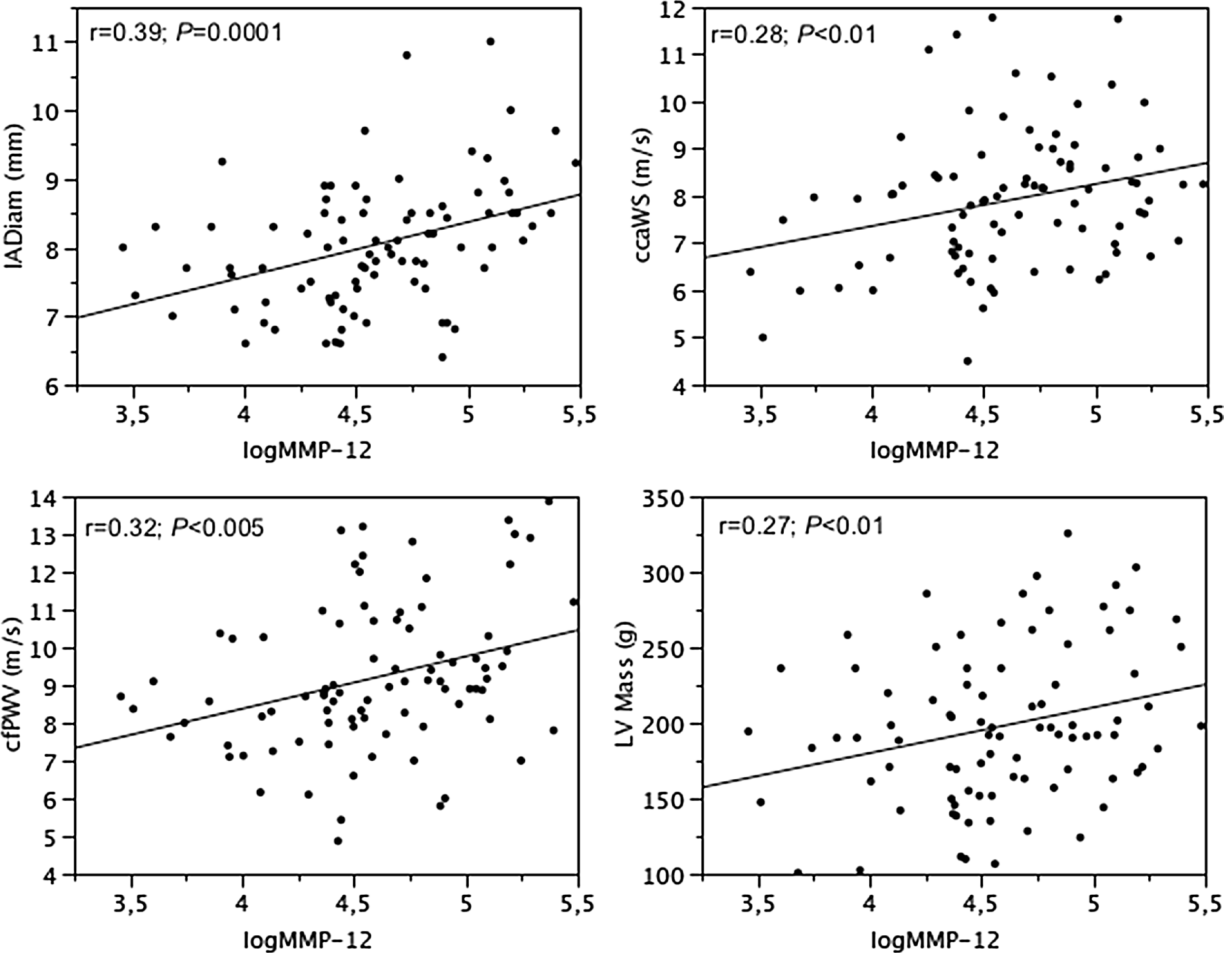
Relationships between MMP-12 and carotid diameter, carotid wave speed, carotid-femoral pulse wave velocity and LV mass in nonT2DM subjects

In nonT2DM subjects, LV mass was related inversely (r = − 0.37; *P *< 0.0005) and e′ and s′ velocities were related directly to HDL (r = 0.34 and 0.26; *P *= 0.001 and 0.01). IADiam, ccaWS, cfPWV and LV mass were directly related to MMP-12 (Fig. 4). CfPWV was also related to fasting glucose (r = 0.22; *P *< 0.01).

In both T2DM patients and nonT2DM subjects, cfPWV was directly related to end-systolic LV wall stress (r = 0.32 and 0.26; *P *< 0.0001 and < 0.01), and s′ (r = − 0.25 and − 0.27; *P *< 0.0005 and < 0.01) and e′ (r = − 0.23 and − 0.32; *P *< 0.005 for both) velocities were inversely related to LV mass. Only in T2DM patients, cfPWV was directly related to LV mass (r = 0.26; *P *< 0.0005) and s′ velocity was inversely related to end-systolic wall stress (r = − 0.23; *P *= 0.001).

The independence of associations between glucose, lipids, ILs, MMP-12 and vascular (Table 2) and cardiac (Table 3) measures was tested in multiple regression models with stepwise removal, after adjustment for age, sex, body size, BP, heart rate, smoking habit, BP-lowering and lipid-lowering therapy and, in T2DM patients, also for diabetes duration and antidiabetic treatment. In T2DM patients, HDL was independently related to cIMT and e′ diastolic velocity and MMP-12 was independently related to IADiam, ccaWS, cfPWV and LV mass. S′ velocity decreased with peak systolic wall stress and LV mass. Plasma glucose, HbA1c or TG were not independently related to CV measures (Tables 2 and 3). In nonT2DM subjects, CV measures were predominantly determined by age, sex and hemodynamic parameters; only IADiam was related to MMP-12 and s′ and e′ velocities were related to HDL (Tables 2 and 3).

**Table 2:** Determinants of vascular biomarkers in T2DM patients and in nonT2DM subjects

	cIMT (μm)		IAD (mm)		ccaWS (m/s)		cfPWV (m/s)	
	T2DM	nonT2DM	T2DM	nonT2DM	T2DM	nonT2DM	T2DM	nonT2DM
	β ± SE		β ± SE		β ± SE		β ± SE	
Sex (male)		0.20 ± 0.09	0.17 ± 0.07	0.37 ± 0.09				
Age (years)	0.22 ± 0.07	0.46 ± 0.09				0.32 ± 0.09		0.58 ± 0.07
Systolic BP (mmHg)	0.15 ± 0.06		0.17 ± 0.06		0.26 ± 0.06	0.40 ± 0.09	0.30 ± 0.06	0.25 ± 0.07
HDL (mmol/L)	− 0.33 ± 0.07							
BP therapy (yes)				0.20 ± 0.09			0.24 ± 0.07	
logMMP-12			0.36 ± 0.06	0.29 ± 0.09	0.27 ± 0.06		0.17 ± 0.06	
R^2^	0.17	0.25	0.22	0.32	0.18	0.26	0.22	0.48
P	< 0.0001	< 0.0001	< 0.0001	< 0.0001	< 0.0001	< 0.0001	< 0.0001	< 0.0001

**Table 3:** Determinants of cardiac biomarkers in T2DM patients and in nonT2DM subjects

	LV mass (g)		s′ velocity (cm/s)		e′ velocity (cm/s)	
	T2DM	nonT2DM	T2DM	nonT2DM	T2DM	nonT2DM
	β ± SE		β ± SE		β ± SE	
Sex (male)		0.31 ± 0.10			0.26 ± 0.08	
Age (years)					− 0.22 ± 0.07	− 0.27 ± 0.09
Body weight (kg)	0.27 ± 0.06	0.32 ± 0.09	0.26 ± 0.07			
Systolic BP (mmHg)	0.20 ± 0.06					
Heart rate (bpm)			0.14 ± 0.06	0.22 ± 0.09		
HDL (mmol/L)				0.24 ± 0.09	0.33 ± 0.07	0.32 ± 0.08
logT2DM duration	0.14 ± 0.06					
Stroke volume (mL)	0.41 ± 0.06	0.21 ± 0.09				
LV mass (g)			− 0.24 ± 0.07		− 0.17 ± 0.07	
LV wall stress (*10^3^ dynes/cm^2^)			− 0.20 ± 0.07			
logMMP-12	0.26 ± 0.05					
R^2^	0.41	0.43	0.19	0.11	0.18	0.21
P	< 0.0001	< 0.0001	< 0.0001	< 0.005	< 0.0001	< 0.0001

### CV organ damage and CV disease

T2DM patients with CV organ damage, i.e. those with cIMT and cfPWV higher than 90th percentiles of the reference population and those with the presence of LV hypertrophy, had significantly lower HDL and higher ILs and MMP-12 than those without organ damage (Table 4), after adjustment for diabetes duration, BP-lowering, lipid-lowering and diabetic treatment, smoking habit and, if applicable, also for sex, age and BP. The two subgroups did not differ for fasting glucose, HbA1c and TG. T2DM patients with CV disease had, as compared to those without CV disease, higher IL-6 and cfPWV (Table 4), after adjustment for sex, age, BP, smoking habit, diabetes duration, BP-lowering, lipid-lowering and diabetic treatment. The two subgroups did not differ for glycemic control, TG, HDL.

**Table 4:** Differences in lipids, ILs and MMP-12 between T2DM patients with and without CV organ damage and differences in ILs and CV measures in T2DM patients with and without CV disease

cIMT (μm)	> 90th percentile (64)	Within limits (127)	*P*
HDL (mmol/L)	1.19 ± 0.25	1.31 ± 0.30	< 0.005
IL-18 (AU)	1499 [790]	1269 [582]	0.01

cfPWV (m/s)	> 90th percentile (52)	Within limits (139)	*P*
HDL	1.13 ± 0.29	1.32 ± 0.27	< 0.0001
MMP-12 (AU)	158 [100]	128 [89]	< 0.05

LVH	Yes (99)	No (92)	*P*
IL-6	38 [28]	34 [23]	< 0.05
MMP-12 (AU)	155 [112]	124 [86]	0.01

CV disease	Yes (97)	No (94)	*P*
IL-6 (AU)	39 [28]	32 [22]	0.01
cfPWV (m/s)	12.1 ± 2.6	10.7 ± 2.1	< 0.05

Within nonT2DM subjects, those with CV events had lower LV systolic longitudinal velocity s′ (8.0 ± 1.2 vs 9.3 ± 1.2 cm/s; *P *= 0.001) as compared to those without events, after adjustment for sex, age, BP, smoking habit, BP-lowering and lipid-lowering treatment.

## Discussion

The present cross-sectional study investigated the relationships between plasma lipids and inflammatory cytokines as well as the impact of lipids and ILs on CV system in T2DM patients. Plasma ILs were directly related to plasma levels of MMP-12 that were inversely related to HDL. MMP-12 was independently associated with carotid diameter, arterial stiffness and LV mass, whereas HDL was independently associated with carotid wall thickness and LV diastolic longitudinal performance.

MMP-12 is a potent elastase highly expressed in macrophages [27]. Diabetes-related metabolic abnormalities, like hyperglycemia, promote macrophage activation and enhances the expression of the inflammatory mediators [28]. Inflammatory cytokines induce MMPs transcription and activation resulting in extracellular matrix alteration [7, 17, 29] that, in the case of MMP-12 activation, is a cleavage of elastin. Elastin is the principal stress-bearing element of large arteries and its fragmentation leads to arterial dilation since arteries cannot withstand anymore the outward forces exerted by arterial pressure [30]. Degradation of elastin contributes also to arterial stiffening. In our T2DM patients, MMP-12 increased with plasma levels of IL-6 and IL-18 and was independently related to elastic artery diameter and stiffness and to LV mass. The association between MMP-12 and LV mass was probably mediated by arterial stiffness as suggested by studies in mice with elastin haploinsufficiency [31].

The role of MMP-12 in arterial wall enlargement and stiffening has been demonstrated in experimental and clinical studies. In MMP-12 knock-out mice, the growth of aortic diameter and the expression of MMP-12 in aortic wall were significantly lower as compared to wild-type mice [32], and an experimental injury of femoral artery did not induce increase in arterial stiffness and MMP-12 mRNA activation that were observed in wild-type mice [33]. In patients with acute dissection of ascending aorta, the plasma levels of MMP-12, IL-6, and IL-8 as well as MMP-12 activity in aortic wall were significantly higher as compared to subjects free of dissection [34, 35].

It is worth to note, that in nonT2DM subjects were observed the same direct associations between ILs and MMP-12 and between MMP-12 and CV measures as in T2DM patients. However, since the levels of ILs and MMP-12 were significantly lower, MMP-12 was independently related only to carotid diameter. This observation is in agreement with results of the Strong Heart Study showing that cardiac target organ damage precedes clinical appearance of T2DM and is related to inflammatory status [36].

Some members of the MMP family, including MMP-12, can degrade the apolipoprotein A-I and decrease circulating HDL levels [8, 37]. In T2DM and nonT2DM populations, plasma MMP-12 was inversely related to plasma HDL levels, which in turn were independently related to mitral longitudinal diastolic velocity e′ in both populations and to mitral longitudinal systolic velocity s′ in nonT2DM subjects. Longitudinal velocities of mitral annulus provide important physiologic information on LV diastolic and systolic performance [25]. In experimental study on anesthetized dogs, an increase in early diastolic velocity e′ during dobutamine infusion was associated with reduction of τ, consistent with faster relaxation, while the reduction in e′ velocity during ischemia was associated with prolongation of τ, consistent with slower relaxation [38]. Similarly, systolic s′ velocity increased during dobutamine infusion in dose-dependent manner and was strongly associated with dP/d*t*_peak_ [39]. A direct association between HDL levels and LV longitudinal systolic and diastolic performance observed in our populations can be explained by studies showing that HDL increases insulin-independent glucose uptake in rat cardiomyocytes via an Akt signaling pathway [40] and that HDL mimetic peptide improves LV diastolic dysfunction in cholesterol-fed rabbits [41].

In T2DM patients, HDL was also independently and inversely related to cIMT. This association has been already described in a large meta-analysis including 21.000 subjects [14] and is probably related to the fact that HDL is able to suppresses smooth muscle cells migration and proliferation [42].

The combined role of ILs, MMP-12 and HDL in CV organ damage of T2DM patients was confirmed also by our observation that diabetic patients with established organ damage, i.e. with increased carotid IMT, aortic stiffness and LV hypertrophy, had significantly lower plasma levels of HDL and higher levels of ILs and MMP-12 as compared to those without vascular and cardiac impairment.

The only CV measure associated with the presence of CV disease in T2DM patients was cfPWV [43]. Indeed, increase in aortic stiffness has a wide impact on CV system. First, it reduces a cushioning function of large arteries thus increasing flow pulsatility in sensitive organs like kidney and brain [44, 45]. Second, it anticipates a return of reflected waves to the heart in late systole, thus augmenting the late-systolic LV wall stress. Increase in LV wall stress stimulates adaptive thickening of myocardial fibers and development of LV hypertrophy [46, 47] that may negatively influence LV myocardial performance. In our T2DM patients, with increasing cfPWV increased end-systolic LV stress and LV mass, which both were inversely related to LV longitudinal systolic performance [48]. Interestingly, in nonT2DM subjects, the only CV parameter associated with CV events was LV longitudinal systolic velocity s′, a finding consistent with results of meta-analysis demonstrating that global LV longitudinal strain is an accurate predictor of major adverse cardiac events [49].

### Study limitations

The present study has some potential limitations. First, plasma insulin levels were not measured, and thus the impact of insulin on ILs, MMP-12 and CV biomarkers cannot be evaluated. Second, measuring HDL levels does not accurately depict their composition and functionality, which can be modified in diabetes. Third, LV diastolic performance was evaluated only by early diastolic longitudinal velocity of mitral annulus and no additional echocardiographic parameters related to LV relaxation and compliance were assessed. Finally, we are aware that cross-sectional design of the study cannot clearly elucidate cause-and-effect relationships.

## Conclusions

Results of this cross-sectional study indicate that IL-6 and IL-18 trigger an increase in MMP-12 plasma levels, which in turn may reduce HDL levels and directly modify vascular structure and function, both in T2DM patients and nonT2DM subjects. Alterations in vascular structure and function elicit changes in the heart (Additional file 1: Figure S1). As expected, the levels of inflammatory cytokines, and consequently those of MMP-12, were significantly higher in T2DM patients that showed more prominent impairment of CV structure and function than nonT2DM subjects. Therefore, targeting inflammatory and MMPs’ pathways could be a part of the strategy to prevent and control diabetes-related CV complications [11, 50].

## Additional file

**Additional file 1: Figure S1.** Role of inflammation and dyslipidemia in cardiovascular organ damage of T2DM patients.

## Data Availability

The datasets used and/or analysed during the current study are not publicly available but are available from the corresponding author on reasonable request.

## Abbreviations

T2DM: type 2 diabetes mellitus
CV: cardiovascular
TG: triglycerides
HDL: high density lipoprotein cholesterol
IL: interleukin
MMP: matrix-metalloproteinase
cfPWV: carotid-femoral pulse-wave velocity
cIMT: carotid intima-media thickness
IADiam: inter-adventitial diameter
ccaWS: local carotid wave speed
LV: left ventricle
s′: systolic longitudinal velocity of mitral annulus
e′: early diastolic longitudinal velocity of mitral annulus
