# Supplementary figures and images for: Cardiovascular organ damage in type 2 diabetes mellitus: the role of lipids and inflammation
Source: Cardiovasc Diabetol. 2019 May 10;18:61. doi: 10.1186/s12933-019-0865-6 (PMC6511166; doi:10.1186/s12933-019-0865-6)

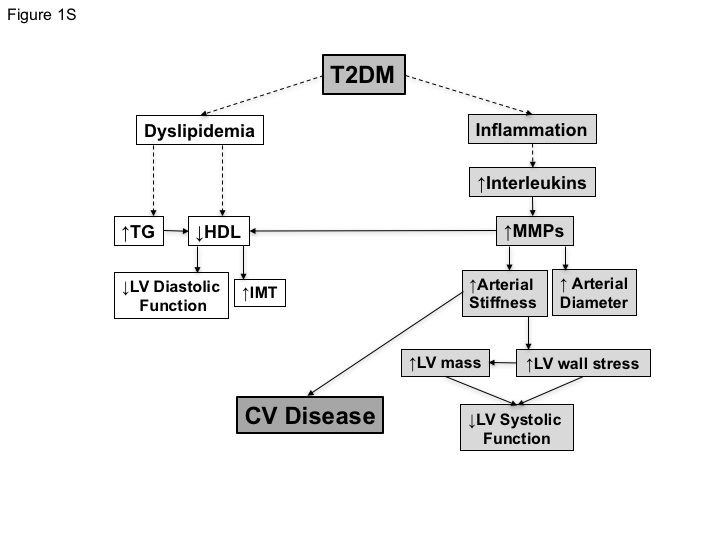

Supplement: Supplementary file 1 — Additional file 1: Figure S1. Role of inflammation and dyslipidemia in cardiovascular organ damage of T2DM patients. [file 12933_2019_865_MOESM1_ESM.tiff]
